# Blood Pressure Continuous Measurement through a Wearable Device: Development and Validation of a Cuffless Method

**DOI:** 10.3390/s21217334

**Published:** 2021-11-04

**Authors:** Beatrice De Marchi, Mattia Frigerio, Silvia De Nadai, Gianluigi Longinotti-Buitoni, Andrea Aliverti

**Affiliations:** 1 Dipartimento di Elettronica, Informazione e Bioingegneria, Politecnico di Milano, 20133 Milano, Italy; gianluigi@x10y.com; 2 L.I.F.E. Italia S.r.l., 20146 Milano, Italy; frigerio.mattia@gmail.com (M.F.); silvia.denadai@x10x.com (S.D.N.)

**Keywords:** blood pressure, continuous monitoring, noninvasive, cuffless, wearable device, photoplethysmography, time-delay method, pulse arrival time

## Abstract

The present study aims to develop and validate a cuffless method for blood pressure continuous measurement through a wearable device. The goal is achieved according to the time-delay method, with the guiding principle of the time relation it takes for a blood volume to travel from the heart to a peripheral site. Inversely proportional to the blood pressure, this time relation is obtained as the time occurring between the R peak of the electrocardiographic signal and a marker point on the photoplethysmographic wave. Such physiological signals are recorded by using L.I.F.E. Italia’s wearable device, made of a sensorized shirt and wristband. A linear regression model is implemented to estimate the corresponding blood pressure variations from the obtained time-delay and other features of the photoplethysmographic wave. Then, according to the international standards, the model performance is assessed, comparing the estimates with the measurements provided by a certified digital sphygmomanometer. According to the standards, the results obtained during this study are notable, with 85% of the errors lower than 10 mmHg and a mean absolute error lower than 7 mmHg. In conclusion, this study suggests a time-delay method for continuous blood pressure estimates with good performance, compared with a reference device based on the oscillometric technique.

## 1. Introduction

Hypertension is a major risk factor for cardiovascular morbidity. It is generally defined according to doctor’s office blood pressure measurements (OBPM) that may not reflect true blood pressure (BP) levels and do not consider diurnal and nocturnal variations.

Nowadays, thanks to the technological development, BP is becoming more and more non-invasively measured by the patient at home (HBPM) or automatically over 24 h (ABPM) [1].

The devices currently employed for HBPM and ABPM usually implement the oscillometric method, which involves an inflatable cuff during the measurement. These devices present several drawbacks:

- Patients may perceive the cuff pressure as being unbearable, especially during sleep, in the case of very high BP and if it is required to frequently repeat the readings;
- Avoiding false elevation or reduction in BP readings and obtaining an accurate measurement require the selection of an appropriate cuff size according to the users’ upper arm circumference;
- Readings by conventional devices may be insufficient indicators for hypertension since only intermittent measurements are provided. These devices cannot record time-varying BP or capture the dynamic state of the cardiovascular system throughout the day.

For these reasons, in the past few years, the interest in developing non-invasive BP measuring devices without an occluding cuff and that is able to return continuous estimations has increased.

### 1.1. Continuous Cuffless BP Measurement Techniques

Cuffless methods are an improvement of the cuff-based ones, designed to overcome their main limitations.

Several continuous cuffless methods were developed in the last years based on the photoplethysmographic (PPG) signal, a simple optical technique that can detect blood volume changes in the microvascular bed of tissues. Examples of methods using this physiological signal are represented by the volume-clamp method, the arterial applanation tonometry method and the time-delay method [1].

#### Time-Delay Method

In the past few years, several research groups have tried to develop continuous cuffless BP measurement techniques based on the guiding principle of the time that a blood volume takes to travel from the heart to a peripheral site, defined as time delay [2,3].

The time delay can be estimated with two different methods [2,4]: the pulse transit time (PTT), based on the time the pulse waveform takes to travel from a proximal to a distal arterial site, and the pulse arrival time (PAT), based on the time the pulse waveform takes to go from the heart to a distal site. This time is defined by the temporal difference between the occurrence of the R-peak in the ECG signal, the ventricular depolarization, and a characteristic point in the PPG waveform.

These two quantities are related as follows:

PAT = PEP + PTT,(1)

where PEP is the pre-ejection period, the time elapsed between the electrical depolarization of the left ventricle (QRS on the ECG) and the beginning of the ventricular ejection. This time represents the period of left ventricular contraction with the cardiac valves closed and its impact on the overall PPT decreases with the distance from the heart. The PEP depends on physiological variables, such as cardiac preload, central arterial pressure, and cardiac contractility. In addition, it changes with stress, physical activity, age, and emotion [5]. The effect of including PEP in the blood pressure estimation is still under investigation [3]. There are studies indicating that BP is less correlated to PAT as compared to PTT, while others state that PAT is a better indicator of BP, as it is dependent on both ventricular contraction and vascular function.

No medical devices using the time-delay method seem to be currently available. After a literature analysis, some prototypal devices have emerged:

- SOMNOtouch NIBP [6], designed by SOMNOmedics, estimates blood pressure through the simultaneous recording of 3-lead ECG signal using wet electrodes and finger PPG signal. Initial calibration with a conventional BP measurement technique is required and its main application is for sleep monitoring.
- CareUp [7] is a smartwatch with an embedded algorithm for blood pressure detection using two different PPG waveforms. One PPG waveform is taken from the back sensor of the watch directly in contact with the wrist skin, and the second is acquired by positioning the index finger of the other hand on the front oximeter sensor. CareUp requires an initial calibration with a conventional BP measurement technique, and the BP estimation is performed intermittently when the subject puts their index finger on the oximeter sensor.
- SeismoWatch [8] is another smartwatch, obtaining blood pressure readings by asking the user to place the upper part of the watch onto their sternum for a short period. An accelerometer inside the watch measures the thoracic vibrations associated with the heartbeat, the seismocardiogram (SCG), to obtain the proximal timing indicating the blood ejection from the left ventricle into the aorta. An optical sensor on the watch measures the photoplethysmographic signal to obtain the distal timing associated with the arrival of the pulse wave at the wrist radial artery. SeismoWatch requires an initial calibration with a conventional BP measurement. The BP estimation is performed intermittently when the subject puts the watch on their sternum.

### 1.2. Aim

This study aimed to develop and validate a non-invasive blood pressure measurement technique that provides continuous blood pressure estimations with a cuffless method.

Among the identified continuous cuffless methods for BP estimation, the pulse arrival time (PAT) was chosen, where the time delay occurring from the R peak of the ECG signal and a characteristic point of the PPG signal is estimated. Necessary physiological signals were acquired thanks to an easy-to-wear wearable device developed by L.I.F.E. Italia S.r.l., consisting of a sensorized shirt and a prototypal wristband to be worn at the radial artery of the left wrist. This kind of device allows the subject to be monitored in daily-life activities without the interference of free cables or finger probes. In addition, the signals acquisition could be continuously performed simply wearing the device with no further actions required to the subject.

From the extracted time-delay indicators, inversely related to blood pressure, linear regression models were implemented and the corresponding blood pressure values were estimated.

The models’ performances was assessed by using dedicated data acquisition protocols designed to evaluate the reliability of the proposed method in detecting both provoked and daily blood pressure variations.

## 2. Materials and Methods

### 2.1. Reference and Test Devices

The reference device was represented by the GIMA ABPM pulse rate monitor [9,10], measuring blood pressure through the oscillometric method. It was equipped with an inflatable cuff connected with an air hose to the measurement instrument. The data and the time of the device were set before the initialization of each acquisition.

The test device was represented by L.I.F.E. Italia’s IIa class medical device, a sensorized shirt that records several physiological signals with a common logic [11,12]:

- A 12-lead ECG signal by using 10 ink-based dry electrodes;
- A 3-channel respiratory signal by using strain circumferential sensors positioned around the body at the thoracic level, xiphoid process, and abdominal level;
- Skin temperature by using a contact sensor near the left armpit;
- Activity level and body position by using one inertial measurement unit (IMU) on the back.

In order to acquire the PPG signal, a prototypal accessory wristband to be wear on the radial artery of the left arm was designed, using the Maxim Integrated MAX30102 optical module [13].

**Figure 1:**
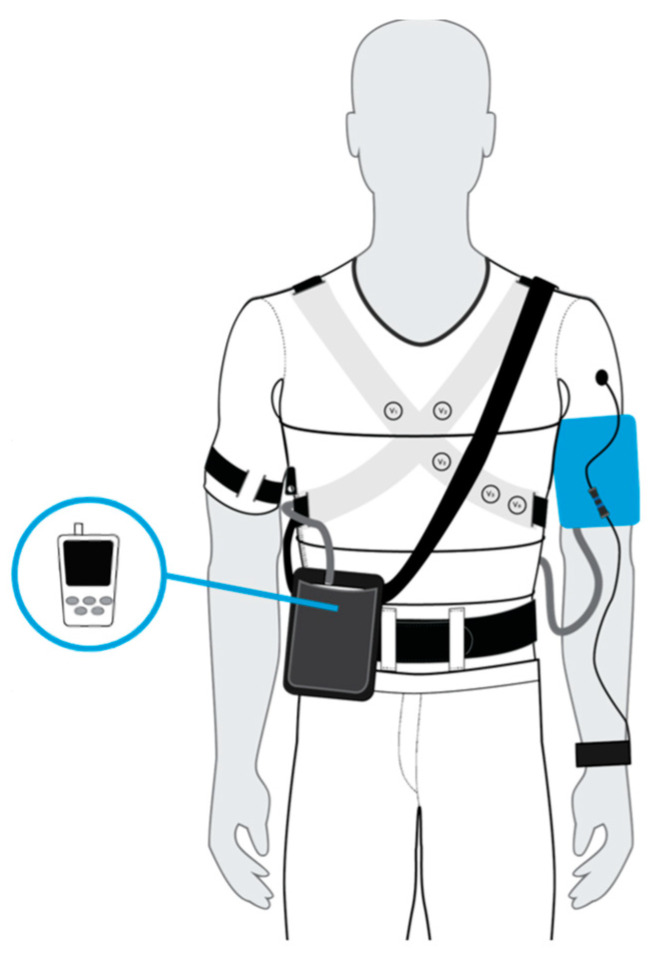
Worn reference and test devices. The reference device inflatable cuff is worn on the left arm and connected with an air hose to the measurement instrument placed in a cross-body bag. The sensorized shirt is worn, thanks to a zipped opening on the side, and sensors adherence is assured with elastic bands. The prototypal wristband is worn on the left wrist, in correspondence of the radial artery and connected to the sensorized shirt with a dedicated connector.

Both the reference device and the wristband had to be worn on the same arm so that the sphygmomanometer occlusion generates a stop in the wristband PPG signal, due to a lack of blood pulsatile motion; this allows an easier synchronization between the two devices. In Figure 1, the device wearing procedure is shown.

### 2.2. Population

The target population consisted of healthy females and males with an age range between 18 and 80 years non presenting with upper limb impairments, dermatological problems or disabling surgery at the acquisition time. The Ethics Committee of Politecnico di Milano approved the study (opinion n. 15; 26 June 2019).

The volunteers that met the pre-screening inclusion and exclusion criteria were enrolled. The enrolled subjects were also asked to fill in an anonymized questionnaire about anthropometrical data and health state. A total of 26 L.I.F.E. Italia’s employees decided to voluntarily participate in the different protocols.

The volunteers involved in the study were asked to provide a written informed consent; then, they were provided with a subject’s specific code known only by the operator dealing with the procedure. The code had the structure of YYMMDDXX, where YYMMDD is the enrollment date and XX is a progressive two-digit number. Characteristics of the involved population are shown in Table 1.

### 2.3. Data Acquisition Protocol

Two acquisition protocols were designed to assess the reliability of the test device in identifying blood pressure variations due to specific stimuli.

- HANDGRIP PROTOCOL
  In order to assess the reliability of the test device against minor blood pressure variations, subjects were called to perform an isometric handgrip (IHG) exercise with the right arm [14]. Three reference blood pressure measurements were performed: one during the rest, one immediately after the exercise, and the last after the recovery. The exercise was repeated on two different days to assess the intrasubject variability.
  The subject had to be seated in rest position for 5 min, and then a blood pressure value was measured with the reference device. The exercise consisted of four 1.5 min contractions of an IHG exercise at 40% maximum effort, with 30 s of recovery periods. For the maximum contraction value determination, the subject was called to perform three grips at the maximum intensity with 10 s rest periods to avoid fatigue; then, the maximum contraction value was given by the average. At the end of the four trials, while the test device was still recording both PPG and ECG signals, the blood pressure was being measured with the reference device. Two minutes after the end of the exercise, with the subject still in rest position, blood pressure was measured with the reference device.
- RUNNING PROTOCOL
  In order to assess the reliability of the test device to more significant blood pressure variations, the subjects were asked to perform running repetitions alternated with walking recovery periods [15] for a total of 10 reference blood pressure measurements immediately after the end of each specific phase. Even in this case, the exercise was repeated on two different days to assess the intrasubject variability.
  The subject had to be seated in rest position for 5 min, and then a blood pressure value was measured with the reference device. After that, the exercise started: 5 min of warm-up walking; 2 min of running at maximum speed; additional 2 min of running at maximum speed; 4 min of recovery walking; 3 min of sprint running; 6 min of recovery walking; 1 min of skipping; 10 min of recovery walking. After each of the aforementioned steps, a BP measurement was performed with the reference device with the subject in a standing position. The subject had to be seated again in the rest position immediately after the recovery walking, and their blood pressure was measured with the reference device. After 2 min in the same position, we proceeded with another measurement.

In addition, in order to assess the reliability of the test device against blood pressure variations not due to specific stimuli, the subjects were asked to perform their daily working activities wearing both the reference and the test devices for at least 5 h:

- DAILY PROTOCOL
  The reference device occlusion occurred automatically every 15 min. At the beginning of each reference device occlusion and for its duration, subjects had to stop and maintain their position to reduce measurement artefacts.
  As an extension of the *daily protocol*, both devices could also be worn during the night to assess the reliability of the test device against the detection of blood pressure variations during sleep. In this case, the reference device occlusion occurred automatically every 15 min.
  Data from the *daily protocol* were valuable to understand the reliability of the test device in a real scenario, continuously monitoring blood pressure during daily activities. For this reason, and for the uncontrolled conditions of the protocol, these data were used only for model test. In addition, real use-case scenario data trends were implemented.

Each subject was required to participate in at least one of the two protocols designed to understand blood pressure variations due to specific stimuli (*handgrip protocol* or *running protocol*). Optionally, each subject could also participate in the protocol designed to identify blood pressure variations in a real scenario (*daily protocol*).

### 2.4. PPG Features

The PPG pulse wave is commonly divided into two phases: the anacrotic phase is the rising edge of the pulse, whereas the catacrotic phase is the falling edge of the pulse [16]. The first phase is primarily concerned with systole, and the second phase with diastole and wave reflections from the periphery. A secondary upstroke is usually present in the catacrotic phase, corresponding to the transient increase in aortic pressure upon closure of the aortic valve, called the dicrotic notch.

According to Refs. [3,16], from each PPG wave, the following marker points were extracted:

- Maximum of the waveform’s first derivative (Md).
- Maximum of the waveform, representing the systolic peak (P).
- Minimum of the waveform, representing the diastolic valley (V).

**Figure 2:**
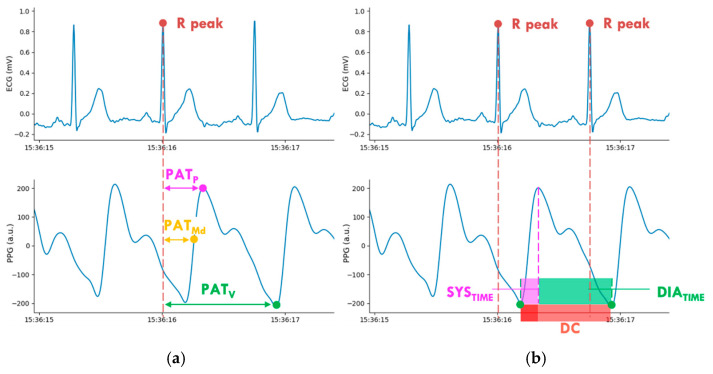
Extracted features from acquired ECG and PPG physiological signals: (**a**) PPG time-delay features; (**b**) PPG morphological features.

Three PPG features were extracted, considering the time delay of each marker point with respect to the previous R peak (Figure 2a):

- Pulse arrival time at the maximum of the waveform’s first derivative (PAT_Md_).
- Pulse arrival time at the systolic peak (PAT_P_).
- Pulse arrival time at the diastolic valley (PAT_V_); it is important to highlight that, for this feature, it is necessary to consider the minimum within the following RR period.

These PPG features, expressed in milliseconds (ms), could be used as time-delay indicators to estimate blood pressure. In order to consider also the PPG wave morphology, additional features were extracted (Figure 2b):

- Systolic time (𝑆𝑌𝑆𝑇𝐼𝑀𝐸): representing the time occurring from the wave start (identified with the previous diastolic valley) to the systolic peak;
- Diastolic time (𝐷𝐼𝐴𝑇𝐼𝑀𝐸): representing the time from the systolic peak to the diastolic valley;
- Duty cycle (DC): representing the ratio of the systolic time and the total PPG wave duration, given by the sum of the systolic and diastolic times ($\frac{SYSTIME\;}{SYSTIME+DIATIME}$).

### 2.5. Features Extraction Algorithm

**Figure 3:**
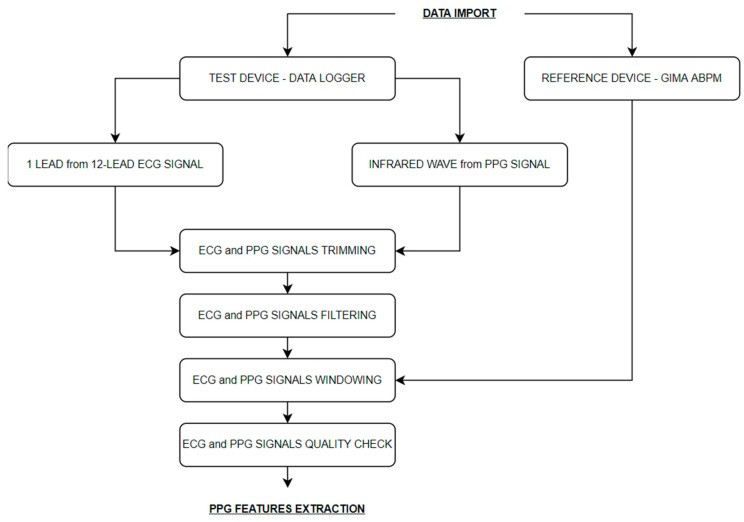
PPG features extraction algorithm flowchart.

The used features extraction algorithm flowchart is summarized in Figure 3. Python Language Reference (version 3.7.0) was used.

Blood pressure values in terms of SBP and DBP were obtained from the reference device with information about the occlusion starting time. The number of collected measurements depend on the adopted protocol.

ECG and PPG signals, imported from the data logger, are continuous signals synchronously recorded by a standard microcontroller at a sampling frequency of 500 Hz and 250 Hz, respectively. A single lead for the ECG signal was selected, in general, the pericardial V6 because it is characterized by the highest quality, and the PPG infrared wave. The ECG signal was then under-sampled at the same PPG frequency (250 Hz) and, if necessary, lower and upper trimmed.

Signals filtering was then performed. The PPG signal passed through a 5th order band pass Butterworth filter with a high pass cutting frequency of 0.5 Hz and a low pass cutting frequency of 10 Hz. The ECG signal passed through a notch filter to remove the 50 Hz interference, and a 5th order band pass Butterworth filter with a high pass cutting frequency of 0.5 Hz and a low pass cutting frequency of 40 Hz.

Given that the reference device provided intermittent measurements, conversely, the test device measurements were continuous, a 30 s window of the test device signals around each reference device measurement was considered in the analysis. 

**Figure 4:**
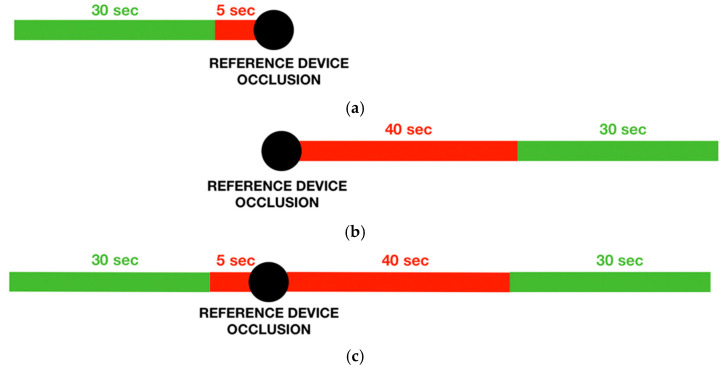
The 30 s window extraction logic: (**a**) previous logic; (**b**) following logic; (**c**) previous and following logic.

According to the different protocols, the 30 s window was extracted with different logic (Figure 4):

- PREVIOUS LOGIC
  The *previous logic* selects a 30 s window before the occlusion starting time, removing the first 5 s to avoid possible artefacts in the PPG wave due to the occlusion. This logic was used for the *handgrip protocol*, in which the subject was in the rest position throughout the protocol, and the stimulus entity was small. Following this logic, it was possible to consider the test device signal during the stimulus occurrence.
- FOLLOWING LOGIC
  The *following logic* selects a 30-s window after the occlusion starting time, removing the first 45 s to wait for a normal PPG signal after the end of the occlusion. This logic was used for the *running protocol*, in which subjects needed to stop the activity before the reference device measurement. Because of the larger entity of the stimulus, also a signal window immediately after the end of the activity could be appropriate.
- PREVIOUS AND FOLLOWING LOGIC
  The *previous and following logic* selects two 30 s windows, one following the *previous logic* and the other one, the *following logic*. This logic was used for the *daily protocol*, in which the subjects’ activity immediately before and after the occlusion could be considered equivalent.

**Figure 5:**
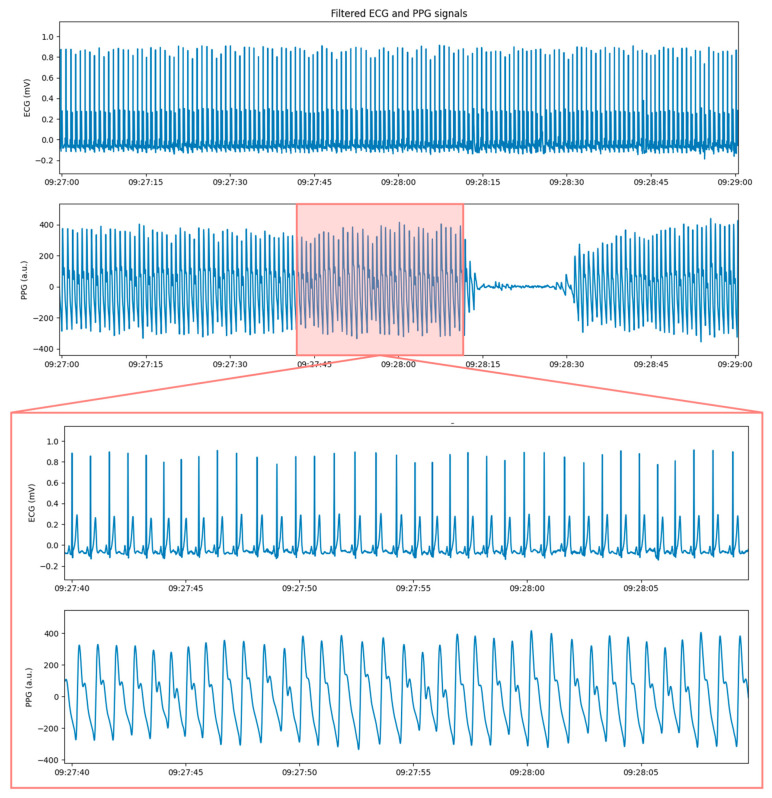
On the top, 2 min window of filtered ECG and PPG signals around the reference device measurement. On the bottom, a zoom on the 30 s window extracted according to the previous logic.

In Figure 5, a 30 s window of filtered ECG and PPG signals extracted around the reference device measurement with the *previous logic*.

From now on, the algorithm considered the 5-s sub-windows of the selected 30 s one at a time.

First of all, for each 5 s window, a signals quality check logic was implemented. The ECG signal quality assessment was performed, using logic related to the signal range, standard deviation (STD) and kurtosis statistical index [17]. In addition, an assessment of the extracted R peaks series was performed, with the hypothesis that within a 5 s window, the heart periods were constant. A good quality PPG signal has a sinusoidal shape with a frequency that matches the heart rate [18]; a bad quality signal deviates from this pattern. As reported in [19], the similarity of the PPG signal to an oscillatory pattern was described using the Hjorth’s parameters, statistical indexes computed in the time domain, but able to provide information on the signal spectrum. Once the R peaks series were validated and the PPG signal passed the Hjorth’s parameters check, we imposed another quality check: physiologically, each sharp rise in the PPG signal has to appear within two consecutive R peaks in the ECG signal. Hence, within two consecutive R peaks, one and only one value for each PPG signal feature is required.

If the quality checks are satisfied, each 5 s window is further spitted into other windows between two consecutive R peaks. Each of these windows has only one PPG wave so that the corresponding features could be extracted.

For each 5 s window, as many values for each feature as the number of R peaks minus one could be obtained. In order to avoid possible outliers, the minimum and maximum values were removed, and an averaging operation was performed. In the end, for each 5 s window, a mean value for each feature was obtained.

**Figure 6:**
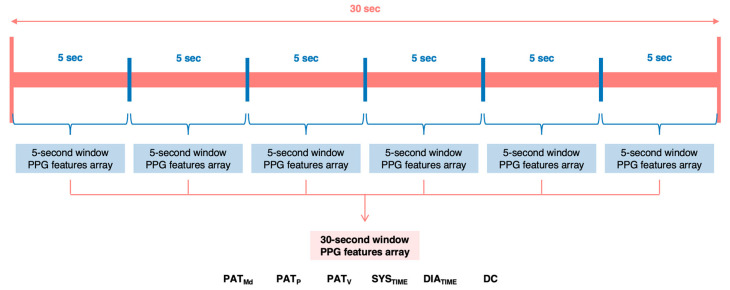
PPG features extraction logic from each 30 s considered window.

All the features’ values, extracted from the same 30 s window, were averaged and collected in a vector with the corresponding reference device values in terms of SBP and DBP. If the windowing logic was *previous and following*, the two vectors from each 30 s window had to be averaged. A summary of the exposed logic is shown in Figure 6.

According to [3], to obtain a blood pressure estimation from the extracted features, a linear regression model was implemented.

As the pulse arrival time at the maximum of the first derivative (PAT_Md_) is the most relevant time-delay feature, it was considered for the statistical data analysis; the necessity of additional features was investigated for the linear regression model implementation. 

## 3. Results

### 3.1. Enrolled Population

Since L.I.F.E. Italia’s employees represent the enrolled population, the currently available data do not include a large variability of age, anthropometric measures, smoke, physical activities, and cardiovascular diseases.

- HANDGRIP PROTOCOL
  For the *handgrip protocol*, 26 subjects (15 men and 11 women) with an average age of 35 (±11 std) years were enrolled. All the enrolled subjects successfully completed the protocol on the first day. Four subjects did not repeat the protocol on the second day because they were unavailable during the second acquisition period. The quality of one subject’s signals was insufficient for the *ECG and PPG signals quality check* block of the features extraction algorithm that was not able to extract good quality windows close to the reference device measurement; therefore, this subject was subsequently removed from the dataset. The low quality, in particular of the PPG signal, can result due to an unsuitable sensor’s position or exerted pressure. The *handgrip protocol* dataset consisted of 25 subjects for the first day and 21 subjects for the second day. The dataset dimension is compliant with the validation study sample size of 20 subjects required by the Institute of Electrical and Electronics Engineers (IEEE) standard [20] for the first phase validation.
- RUNNING PROTOCOL
  For the *running protocol*, three subjects (one man and two women) with an average age of 30 (±6 std) years were enrolled. All the three enrolled subjects successfully completed the protocol on both days, and all the data were included in the dataset.
- DAILY PROTOCOL
  For the *daily protocol*, 20 subjects (12 men and eight women) with an average age of 36 (±11 std) years were enrolled. A total of 12 subjects (six men and six women) successfully completed the protocol, with an acquisition period higher than the required five hours. The other eight subjects had lower acquisition periods due to technical problems during the acquisitions. A 24-year-old woman assessed the reliability of the device also during the night.

### 3.2. Statistical Handgrip Protocol Data Analysis

A preliminary statistical analysis was conducted because of the significant amount of data available for the *handgrip protocol*. In fact, after the outliers’ removal process (see details at the end of this section), the *handgrip protocol* dataset includes 24 subjects for the first acquisition day and 19 for the second acquisition day, for a total amount of 129 features’ vectors.

Due to the time-delay method characteristics, the data were analyzed in terms of differential values, allowing the mitigation of subject-specific dependencies. From the reference device, differential systolic (ΔSBP), diastolic (ΔDBP) and mean (ΔMBP) blood pressure values were considered; from the test device, differential PAT_Md_ feature (ΔPAT_Md_).

In the *handgrip protocol*, three reference blood pressure measurements were performed: one at rest, one immediately after the exercise and the last after a recovery period. Consequently, the following differential values were established:

- The difference between effort and rest values, called ΔEffort: a blood pressure increase and a corresponding PAT_Md_ decrease are expected.
- The difference between recovery and effort values, called ΔRecovery: a blood pressure decrease and a corresponding PAT_Md_ increase are expected.
- The difference between recovery and rest values, called ΔRest: these two values are expected to be very similar, so the difference needs to be around zero.

**Figure 7:**
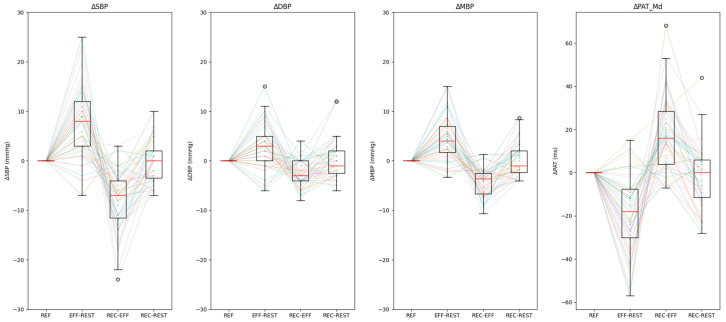
Variation of Systolic Blood Pressure (ΔSBP), Diastolic Blood Pressure (ΔDBP), Mean Blood Pressure (ΔMBP) and Pulse Arrival Time (ΔPAT) in the different protocol phases: ΔEffort, ΔRecovery and ΔRest. Individual subjects’ values are reported as colored dotted lines with overlaid boxplots (lower whisker: Q1 − 1.5 * IQR; upper whisker: Q3 + 1.5 * IQR).

A characteristic *V-shape* is expected for all the considered variables: in physiological conditions, the blood pressure increases after the exercise, then decreases in recovery and returns to rest values after recovery. Conversely, according to the literature evidence [3], the PAT_Md_ behaves oppositely: it decreases after having completed the exercise and increases during the recovery. In Figure 7, the attended V-shape is shown with boxplots and individual subjects’ values.

**Figure 8:**
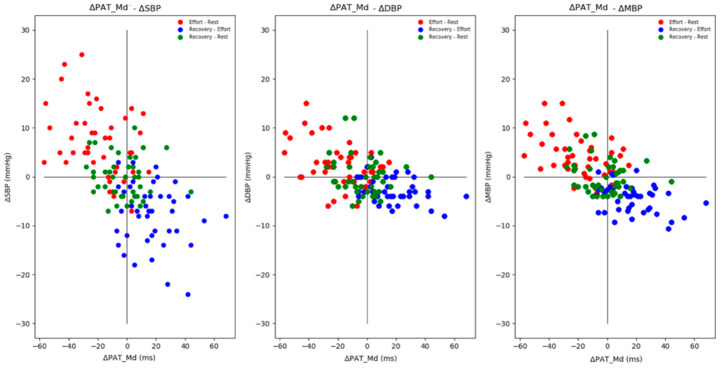
Handgrip protocol data ΔPAT_Md_ relation with ΔSBP (**left**), ΔDBP (**center**) and ΔMBP (**right**) in rest (green), effort (red) and recovery (blue) phases.

In Figure 8, the *handgrip protocol* dataset scatter plot is shown to understand the relation between test device values, in terms of PAT_Md_ variation, and reference device values, in terms of systolic (SBP), diastolic (DBP) and mean (MBP) blood pressure variation. In Table 2, the corresponding Pearson’s correlation coefficients and *p*-values are shown.

As expected, ΔEffort values (in red) fell into the second quadrant, corresponding to an increase in blood pressure and a decrease in PAT_Md_ values. Symmetrically, ΔRecovery values (in blue) fell into the fourth quadrant, in which the blood pressure drops and PAT_Md_ increases. Finally, the ΔRest values (in green) were centered around zero. In Table 3, the dataset median and IQR for each protocol phase are shown.

The *handgrip protocol* dataset’s outliers removal process had the aim of removing the subject’s acquisitions in which the majority of the three recorded values fell in the wrong quadrants. According to this logic, both the acquisitions of one subject and the second day acquisition of another subject were removed.

### 3.3. Statistical Training Dataset Analysis

The training dataset included the *handgrip protocol* and *running protocol* data for most significant blood pressure variations identification. The selected dataset consisted of 174 feature vectors: 129 from the *handgrip protocol* dataset and 45 from the *running protocol* dataset after the outliers’ removal process. In the *running protocol*, values were expected to fall in the second quadrant or to be close to zero. In fact, both during the exercise and the recovery periods, blood pressure values higher than the ones in rest position were expected. For this reason, data coming from the first day acquisition of one subject (a 24-year-old woman) and falling in the first quadrant were removed.

Despite being aware of the unbalancing between the two datasets, the *running protocol* data inclusion was essential to cover larger BP variations. In addition, since the proposed model is based on features, the 10 values for each feature coming from the *running protocol* acquisitions allowed us to consider consistently the available data amount.

**Figure 9:**
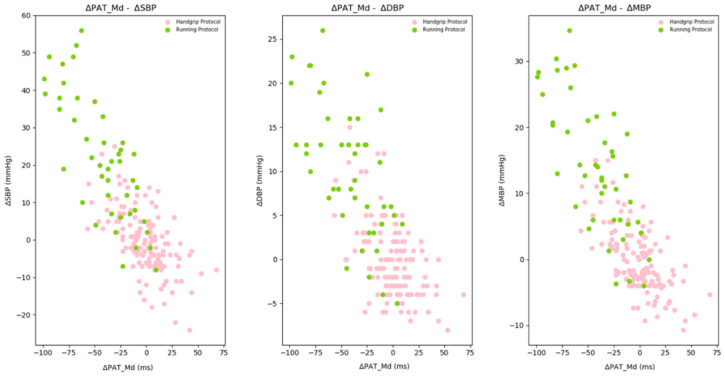
Training dataset ΔPAT_Md_ relation with ΔSBP (**left**), ΔDBP (**center**) and ΔMBP (**right**). Pink dots come from the handgrip protocol dataset; green dots come from the running protocol dataset.

Including also the *running protocol* data, according to the physiological inverse linear relation between blood pressure and time-delay [3], the training dataset was enriched with significant PAT_Md_ variations in the negative direction. In Figure 9, the training dataset scatter plot is shown, with the *handgrip protocol* in green and *running protocol* in pink; the corresponding Pearson’s correlation coefficients and *p*-values are shown in Table 4.

### 3.4. Linear Regression Model for Blood Pressure Estimation

For each blood pressure value to be estimated (systolic, diastolic, and mean), a specific multiple linear regression model was implemented, including PPG features with a significant correlation with blood pressure (Pearson’s correlation coefficient ≥ 0.60). As shown in Table 5, all blood pressure values presented the highest correlation with the same three PPG features: PAT at the maximum of the first derivative (PAT_Md_), PAT at the diastolic valley (PAT_V_) and diastolic time (DIA_TIME_).

Implemented models with slopes *a*, *b* and *c* required null intercepts because dealing with differential values. Otherwise, a blood pressure change was estimated, even for no features variation:

ΔSBP = a_SBP_ * ΔPAT_Md_ + b_SBP_ * ΔPAT_V_ + c_SBP_ * ΔDIA_TIME_ (2)

ΔDBP = a_DBP_ * ΔPAT_Md_ + b_DBP_ * ΔPAT_V_ + c_DBP_ * ΔDIA_TIME_ (3)

ΔMBP = a_MBP_ * ΔPAT_Md_ + b_MBP_ * ΔPAT_V_ + c_MBP_ * ΔDIA_TIME_ (4)

### 3.5. Models’ Performances Assessment

The performance of the proposed multiple linear regression models was assessed through a 10-folds cross validation. The training dataset was iteratively used $\frac{9}{10}$ for model training and $\frac{1}{10}$ for model validation; each tenth of the dataset occurs only one time as the validation set.

The obtain results were evaluated, according to the IEEE standard [20] for cuffless blood pressure wearable devices, requiring the involvement of at least 20 subjects in a first validation phase: report of the number of BP errors (predicted-measured) was within 5, 10 and 15 mmHg; at least 85% of the BP errors were within 10 mmHg; the mean absolute error (MAE) was less than 7 mmHg in the first validation phase; the Bland–Altman plot was used as a graphical method.

**Figure 10:**
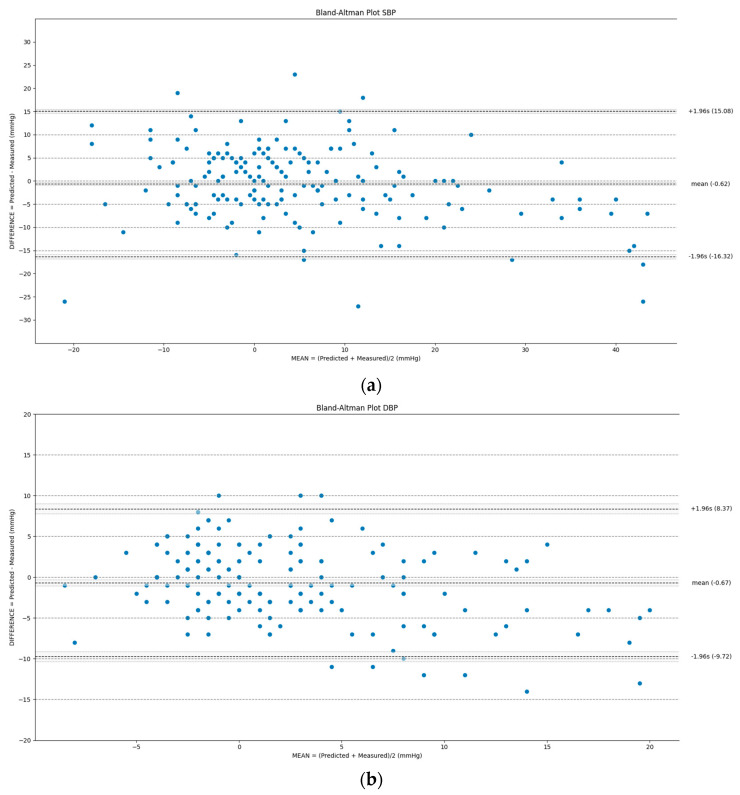

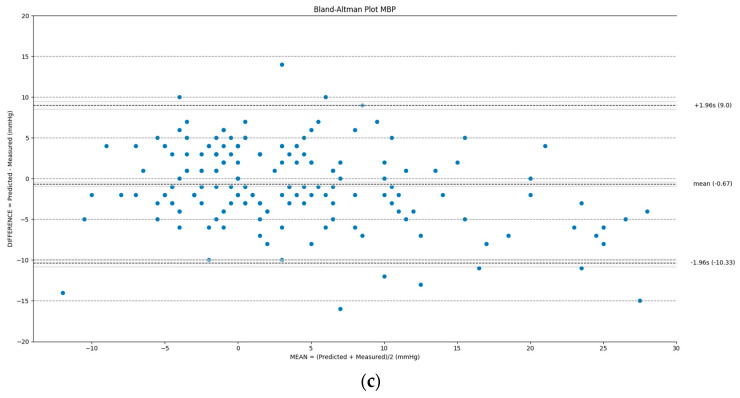
Bland–Altman plots for the multiple linear regression proposed models: (**a**) SBP; (**b**) DBP; (**c**) MBP.

- SBP MODEL
  For the SBP model, 84.0% of the errors was under 10 mmHg (Table 6), and the MAE was 6 mmHg. From the corresponding Bland–Altman plot (Figure 10a), the model error did not seem dependent on a specific mean value. No significant bias was present: the average of the differences between the paired data was −0.62 mmHg. In addition, more than 94% of the data were within both the 15 mmHg and the 1.96 s ranges.
- DBP MODEL
  For the DBP model, 96.5% of the errors were less than 10 mmHg (Table 6), and the MAE was 4 mmHg. The 1.96s ranges corresponded with the 10 mmHg ones, and over 96% of the data were within them. The corresponding Bland–Altman plot is shown in Figure 10b.
  No significant bias was present: the average of the differences between the paired data was −0.67 mmHg. Even in this case, no relationship seems to be present between the differences and mean values. However, if high differences were recorded, these were negative and correlated with significant and positive blood pressure variations.
- MBP MODEL
  For the MBP model, 95.4% of the errors were less than 10 mmHg (Table 6), and the MAE was 4 mmHg. The 1.96 s ranges corresponded with the 10 mmHg ones, and over 93% of the data were within them. No significant bias was present: the average of the differences between the paired data was −0.67 mmHg. The corresponding Bland–Altman plot is shown in Figure 10c. Even in this case, no relationship seems to be present between differences and mean values.

### 3.6. Models’ Test

As a test set, data collected with the *daily protocol* were used, considering differential values with respect to the first acquired value of each subject.

Since the blood pressure measurements of this protocol were not performed in controlled conditions, these data did not appear adequate for the model training. However, the idea is that a regression model, trained on a dataset in which a wide range of variation in terms of PAT_Md_ and blood pressure is present, can be then used to estimate blood pressure values even when recorded during the usual subjects’ activities and not only if due to specific stimuli.

In order to reduce possible oscillations in the data coming from measurement artefacts, a moving average with a sliding window of 4 samples and a stride of one was performed. This window’s size dimension was used to have a mean blood pressure value of each acquisition hour, considering that the reference device performs a measurement every 15 min.

As was done for the training phase, the models’ test performances were assessed according to the IEEE standard requirements for cuffless blood pressure measurement devices based on errors ranges, mean absolute errors and Bland–Altman plots.

The first requirement of having at least 85% of the errors within the 10 mmHg threshold was passed by the DBP and MBP models. Conversely, only 80.0% of the errors were within the range for the SBP model. All the considered models passed the second requirement of a MAE less than 7 mmHg.

More than 90% of the data were within the 15 mmHg and the 1.96 s ranges for the SBP model. The 1.96 s ranges correspond with the 10 mmHg ones, and more than 92% of the data are within them for the DBP and MBP models.

### 3.7. Real Use Case Scenario

The considered multiple regression models are suitable for estimating blood pressure variation exploiting a series of time-delay features from preliminary analyses. 

Blood pressure variations provided by the models (ΔBP) can be used to obtain corresponding absolute blood pressure values (BP_estimated_) starting from the initial calibration value (BP_reference_) to reproduce a real use case scenario:

SBP_estimated_ = SBP_reference_ + ΔSBP (5)

DBP_estimated_ = DBP_reference_ + ΔDBP (6)

MBP_estimated_ = MBP_reference_ + ΔMBP (7)

**Figure 11:**
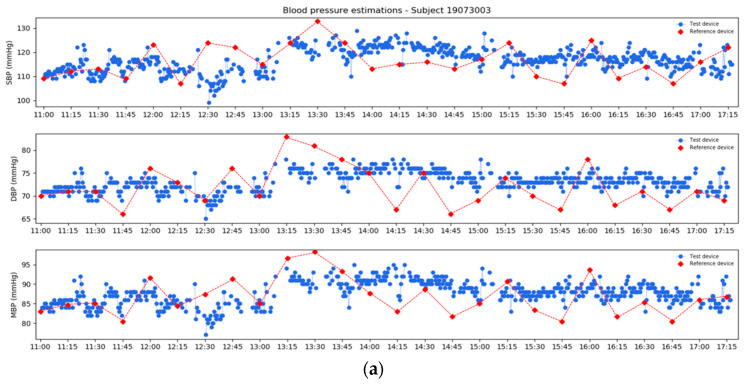

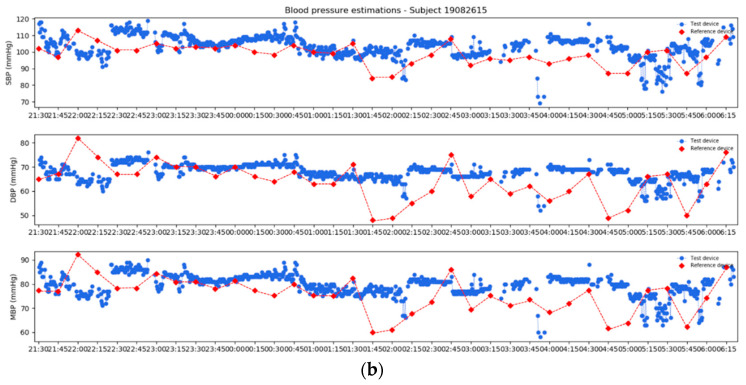
Estimated profiles (in blue) compared with reference device values (in red): (**a**) daytime; (**b**) nighttime.

In Figure 11, estimated daytime and nighttime profiles (in blue) are compared with reference device values (in red). For data comparison, it has to be considered the uncontrolled conditions of the daily protocol, also reflected in higher instability of the reference device measurements.

In the daytime example, data were acquired for a period of six hours during a working day at the office: in the first part, the subject was involved in working activities at a desk, followed by an hour and a half of a lunch break, from 12:30 p.m. to 2:00 p.m., and again working activities at a desk. A total of 26 measurements were returned by the reference device, occluding every 15 min; conversely, the test device provided a BP estimation every 30 s for a total amount of 756 measurements. The *ECG and PPG signals quality check* block of the proposed features extraction algorithm identified as low quality only the 20.8% of the considered windows. This corresponds to no BP estimations and missing values in the plot.

In the nighttime example, data were acquired for about a period of nine hours: in the first part, the subject was awake; their sleeping period started at 11:00 p.m. and ended about 5:30 a.m. A total of 36 measurements were returned by the reference device, occluding every 15 min even in this case; conversely, the test device returned a total amount of 1081 measurements. In the 16.5% of the considered windows, the algorithm missed the estimation for low signal quality.

## 4. Discussion

In this study, we proposed a continuous cuffless blood pressure estimation method, evaluating performances according to the IEEE standard for wearables devices [20]. The models’ performance, verified on the training set through the use of cross-validation, met the standards. The diastolic and mean blood pressure models satisfied both requirements, with 96.5% and 95.4% errors below 10 mmHg and a MAE of 4 mmHg. The systolic blood pressure model showed 84.0% errors below 10 mmHg, slightly below the threshold, and a MAE of 6 mmHg.

Testing the models’ performances on the data collected through the *daily protocol*, all the models presented a MAE of 6 mmHg. In the diastolic and mean blood pressure models, 87.7% of the errors were below 10 mmHg, while in the systolic model, only 80.0%.

### 4.1. Limits

The principal limit of the proposed method is the necessity of an initial calibration measurement and, consequently, the models’ performance is strictly dependent on the quality of this measurement. Before starting the acquisition with L.I.F.E. Italia’s wearable device, the reference measurement could be recorded with a certified sphygmomanometer, repeated to increase the estimation accuracy. However, even in the literature [3], no time-delay methods for blood pressure estimation without an initial calibration measurement seem to be now available.

Another critical aspect is represented by the PPG signal quality, which is strictly dependent on the prototypal wristband adherence and position in correspondence with the radial artery. In addition, avoid wrist obstruction and maximize user comfort are crucial elements.

Additional subjects are required for models’ performances improvement, in particular for the *running protocol* dataset, currently made of only three subjects. In addition, since the model test was performed with data coming from the same population of the training dataset but involved in a different protocol, additional subjects from a totally independent sample are required to strengthen the results.

Finally, the actually enrolled population is represented by L.I.F.E. Italia’s employees, healthy adults characterized by an early average age; a more diverse population could allow to deeper access the reliability of the proposed method.

### 4.2. Strengths

The main strength of the proposed method is represented by the acquisition technology, designed to record several physiological signals with a common logic and each one with its proper sampling frequency: these two elements are relevant for the implementation of a time-delay based method, such as the proposed one.

In addition, the abundance of information coming from a 12-lead ECG signal allows to select the lead characterized by the highest signal quality. Furthermore, using a wearable technology able to record several physiological signals in daily context allows proposing more complex monitoring scenarios.

According to the feedback collected in a non-structured way at the end of the protocols, the test device was well tolerated both during the *handgrip* and *running protocols*, designed to assess the reliability of the device in identifying blood pressure variations due to specific stimuli, and during daily-life activities proposed in the *daily protocol*; as expected, the necessity of optimizing the prototypal wristband for a comfort improvement emerged.

Another strong point of the proposed method is the protocols, designed to provoke specific blood pressure variations but always in a physiological range. This can help demonstrate the suitability of the proposed method for real-life contexts, even if could lead to lower model performance.

### 4.3. Future Developments

The obtained performance could be improved to increase the models’ accuracy and reduce dependency on calibration measurements. 

The first improvement could be focused on the prototype optimization in terms of optical components, sensors case, textile components and positioning on the body of the module. These improvements will increase the obtained PPG signal quality and the comfort.

Models’ performances improvement could be performed by enlarging the *running protocol* dataset, used in combination with the *handgrip protocol* dataset for model training. Only three subjects were enrolled so far in the *running protocol*, against more than 20 subjects of the *handgrip protocol*.

The inclusion of additional features is another strategy for proposed linear regression models improvement. These can be represented by a series of qualitative features available in the questionnaire filled in by each subject involved in the protocol: sex, age, anthropometric measures, smoke, physical activities, and cardiovascular diseases. The inclusion of these data could also show the necessity of specific cluster-based models, i.e., splitting the dataset into homogeneous classes and implementing different models, each one best fitting a specific class. The currently available data did not include a large variability of these features; therefore, it did not seem helpful in exploiting them in the model.

The availability of a multi-parametric acquisition platform allowed to synchronously record several physiological signals in addition to the ones strictly necessary for the BP estimations. As a future development, it could be interesting to analyze the correlation of BP values with the others available physiological signals (i.e., respiration, activity level).

Being that the proposed regression models are linear, their performances could be improved by implementing a more complex, higher-order model.

Finally, only healthy subjects were enrolled so far: dedicated clinical trials could allow investigating the proposed method adequacy in the case of hypertension or related pathologies.

In conclusion, the results of the study are notable because it is proposed a time-delay method for blood pressure estimations with good performance, compared with a reference device based on the oscillometric technique. In a real-case scenario, through L.I.F.E. Italia’s wearable device and thanks to the proposed cuffless method for blood pressure variations estimation, subjects could monitor their blood pressure values without the inconvenience of a sphygmomanometer.

## Figures and Tables

**Table 1:** Involved population characteristics as derived from the filled questionnaires.

	All (26 Subjects)	Men (15 Subjects)	Women (11 Subjects)
	Mean ± Std	Mean ± Std	Mean ± Std
Age (years)	35 ± 11	38 ± 12	34 ± 9
Height (cm)	173 ± 7.26	177 ± 6.40	168 ± 3.45
Weight (kg)	67 ± 8.99	72 ± 6.10	61 ± 8.40
BMI (kg/m^2^)	22 ± 2.30	23 ± 1.76	22 ± 2.80
Smoking	3 out of 26	2 out of 15	1 out of 11
Regular physical activity	19 out of 26	10 out of 15	9 out of 11

**Table 2:** Pearson’s correlation coefficients and *p*-values of the relation between ΔPAT_Md_ and ΔSBP, ΔDBP and ΔMBP for handgrip protocol data.

	r	*p*-Value
ΔSBP and ΔPAT_Md_	−0.61	1.98 × 10^−14^
ΔDBP and ΔPAT_Md_	−0.53	8.82 × 10^−11^
ΔMBP and ΔPAT_Md_	−0.65	3.67 × 10^−17^

**Table 3:** Handgrip protocol data ΔPAT_Md_ relation with ΔSBP, ΔDBP and ΔMBP in terms of median and IQR.

	ΔPAT (ms)	ΔSBP (mmHg)	ΔDBP (mmHg)	ΔMBP (mmHg)
	Median ± IQR	Median ± IQR	Median ± IQR	Median ± IQR
Effort–Rest	−18.0 ± 22.5	8.0 ± 9.00	3.0 ± 5.00	4.0 ± 5.33
Recovery–Effort	16 ± 24.50	−7.0 ± 7.50	−3.0 ± 4.00	−3.67 ± 4.16
Recovery–Rest	0.0 ± 17.50	0.0 ± 5.50	−1.0 ± 4.50	−1.0 ± 4.33

**Table 4:** Pearson’s correlation coefficients and *p*-values of the relation between ΔPAT_Md_ and ΔSBP, ΔDBP and ΔMBP training dataset.

Variables	r	*p*-Value
ΔSBP and ΔPAT_Md_	−0.80	4.26 × 10^−40^
ΔDBP and ΔPAT_Md_	−0.73	1.52 × 10^−30^
ΔMBP and ΔPAT_Md_	−0.81	3.21 × 10^−41^

**Table 5:** PPG features correlation with SBP, DBP and MBP.

	SBP	DBP	MBP
	r (*p*-Value)	r (*p*-Value)	r (*p*-Value)
PAT_Md_	−0.80 (<0.001)	−0.73 (<0.001)	−0.79 (<0.001)
PAT_P_	−0.46 (<0.001)	−0.50 (<0.001)	−0.55 (<0.001)
PAT_V_	−0.78 (<0.001)	−0.72 (<0.001)	−0.82 (<0.001)
SYS_TIME_	−0.07 (=0.33)	−0.13 (=0.10)	−0.16 (=0.03)
DIA_TIME_	−0.75 (<0.001)	−0.65 (<0.001)	−0.75 (<0.001)
DC	0.45 (<0.001)	0.35 (<0.001)	0.39 (<0.001)

**Table 6:** Number and percentage of differences within the ranges of 5, 10 and 15 mmHg for the multiple linear regression proposed models.

	SBP Model	DBP Model	MBP Model
	# of Values (%)	# of Values (%)	# of Values (%)
≤5 mmHg	99 out of 174 (56.9%)	135 out of 174 (77.6%)	135 out of 174 (77.6%)
≤10 mmHg	146 out of 174 (84.0%)	168 out of 174 (96.5%)	166 out of 174 (95.4%)
≤15 mmHg	164 out of 174 (94.25%)	174 out of 174 (100%)	173 out of 174 (99.4%)

## Data Availability

Data are available at L.I.F.E. Italia S.r.l.’s data repository.
